# Supplementary figures and images for: The impact of viral and host factors on the influenza A virus transmission bottleneck
Source: PLoS Pathog. 2026 Mar 25;22(3):e1014079. doi: 10.1371/journal.ppat.1014079 (PMC13038104; doi:10.1371/journal.ppat.1014079)

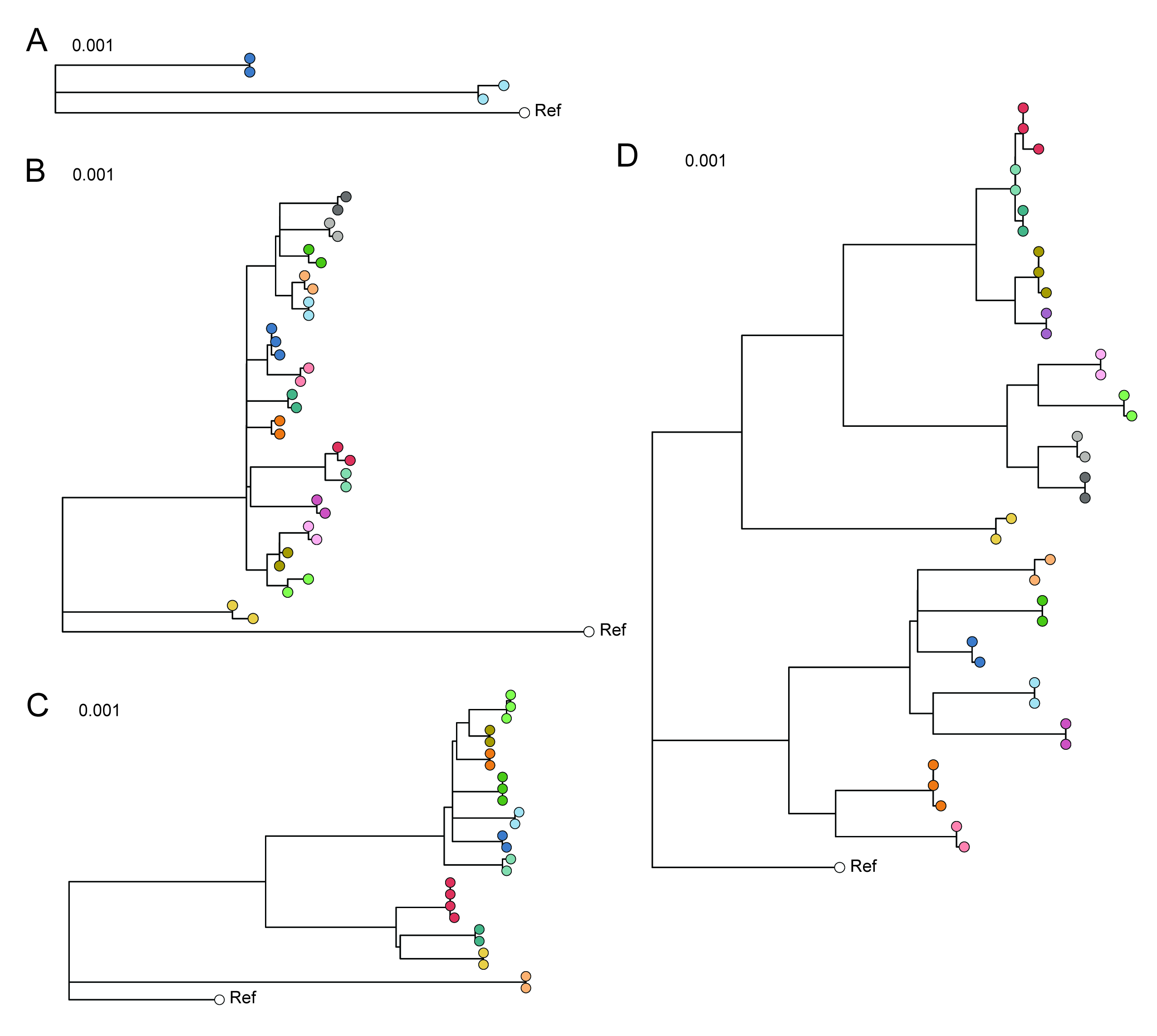

Supplement: S1 Fig — (A-D) Phylogenetic trees of all samples. Tips are colored based on household membership with colors in separate trees representing non-related households. Individual panels represent unique reference strains as follows: (A) A/Michigan/2017 A(H1N1)pdm09, (B) A/Singapore/2018 A(H3N2), (C) A/Brisbane/2019 A(H1N1)pdm09, (D) A/Darwin/2021 A(H3N2). (TIF) [file ppat.1014079.s001.tif]

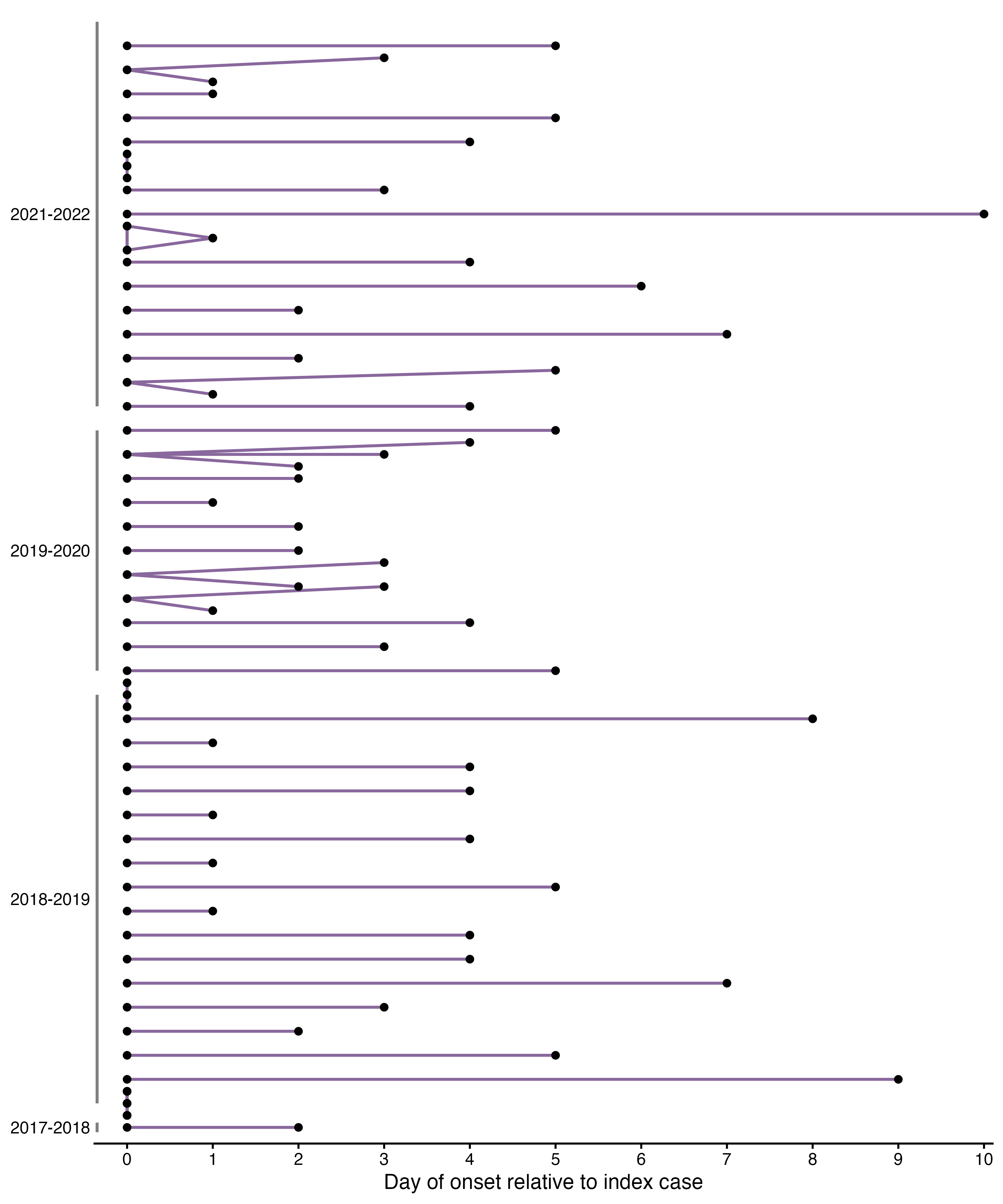

Supplement: S2 Fig — Dots represent all individuals included in our final analysis set. Lines connect members of a transmission pair. Individuals with the earliest symptom onset date are defined as the index case. In cases where two individuals in a single household have an earliest-for-household onset date, both cases are assigned as index cases. (TIF) [file ppat.1014079.s002.tif]

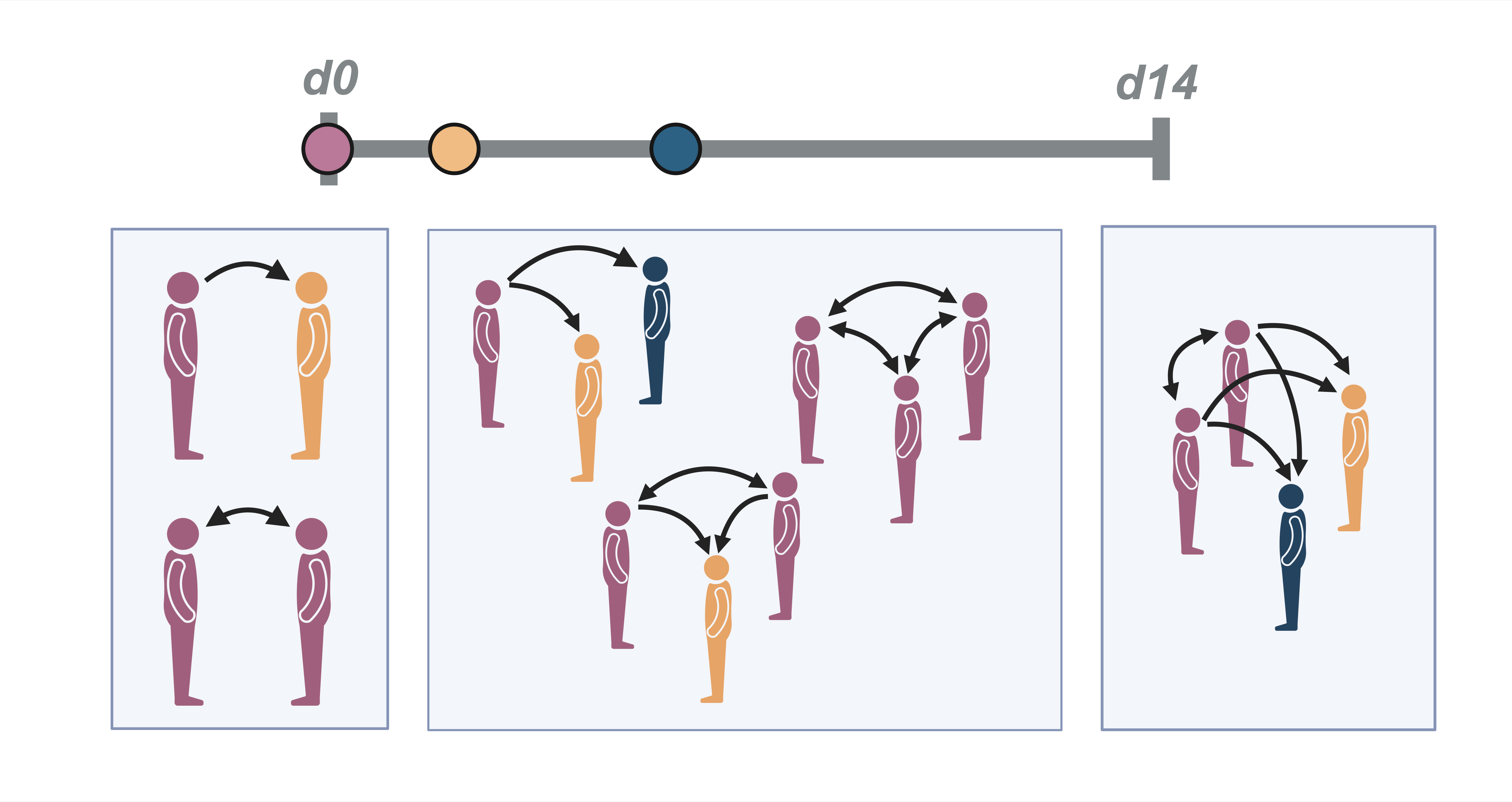

Supplement: S3 Fig — Individual samples are assigned into pairs based on the date of symptom onset. Each arrow indicates a transmission pairing with the arrowhead pointing towards a recipient. Created in BioRender. Krupinsky, K. (2026) https://BioRender.com/q34qakq. (TIF) [file ppat.1014079.s003.tif]

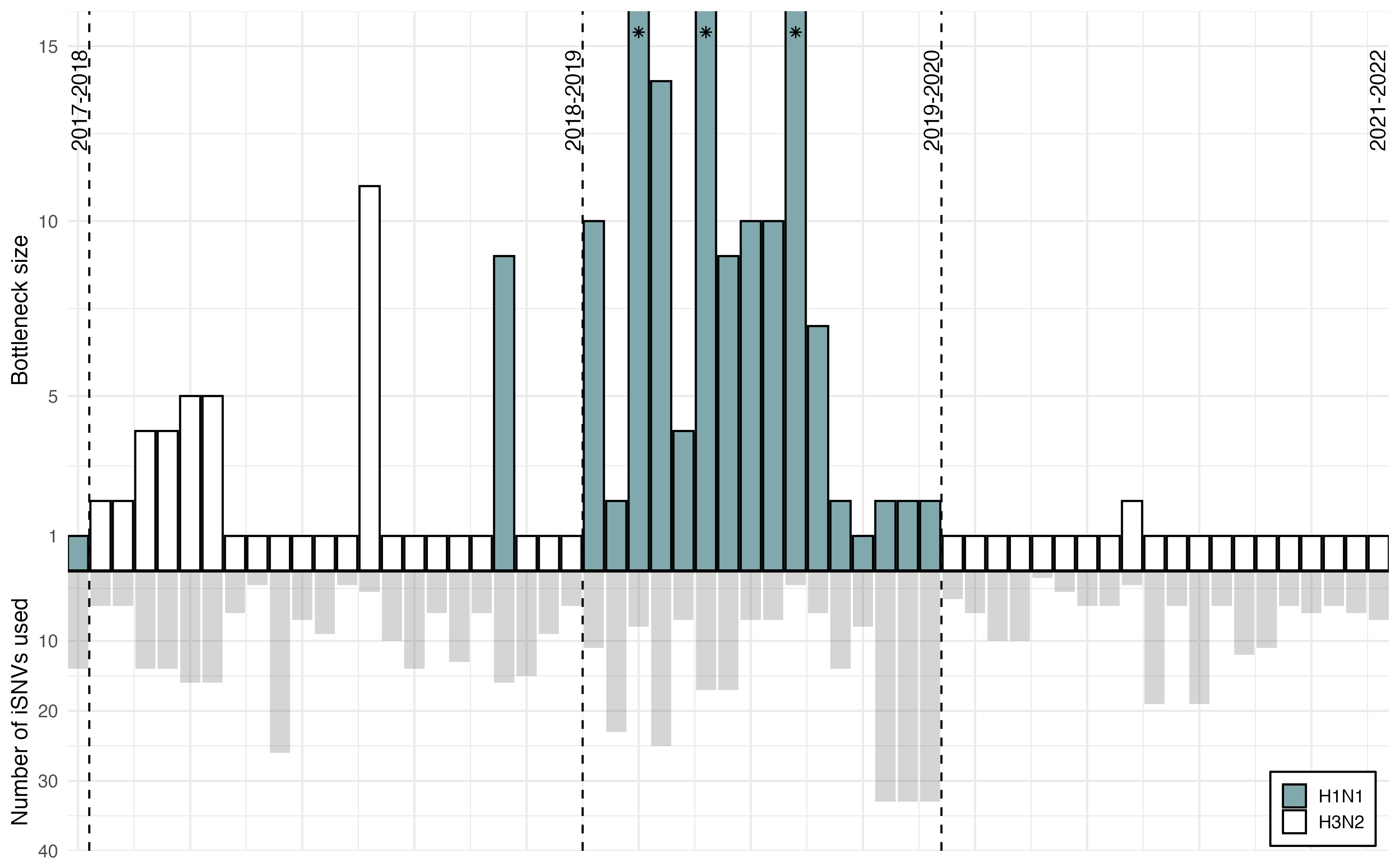

Supplement: S4 Fig — Bottleneck estimates for individual transmission pairs using beta-binomial model (top) and number of iSNVs used in each estimate (bottom). Filled in (teal) bars represent pairs with A(H1N1)pdm09; open (white) bars represent pairs with A(H3N2). iSNVs were used if they were found in the donor pair-member or both pair-members (i.e., iSNVs were not used if found exclusively in recipient pair-member). Stars on bars represent maximum likelihood estimates greater than 15. (TIF) [file ppat.1014079.s004.tif]
